# An epidemiologic study comparing cancer- and noncancer-associated venous thromboembolism in a racially diverse Southeastern United States county

**DOI:** 10.1016/j.rpth.2024.102420

**Published:** 2024-04-26

**Authors:** Andrew M. Peseski, Sargam Kapoor, Maragatha Kuchibhatla, Alys Adamski, Karon Abe, Michele G. Beckman, Nimia L. Reyes, Lisa C. Richardson, Ibrahim Saber, Ryan Schulteis, Bhavana Pendurthi Singh, Andrea Sitlinger, Elizabeth H. Thames, Thomas L. Ortel

**Affiliations:** 1 Division of Hematology, Department of Medicine, Duke University, Durham, North Carolina, USA; 2 Department of Biostatistics and Bioinformatics, Duke University, Durham, North Carolina, USA; 3 Division of Blood Disorders and Public Health Genomics, Centers for Disease Control and Prevention, Atlanta, Georgia, USA; 4 Division of Cancer Prevention and Control, Centers for Disease Control and Prevention, Atlanta, Georgia, USA; 5 AbbVie Inc, North Chicago, Illinois, USA; 6 Department of Medicine, Duke University, Durham, North Carolina, USA; 7 Division of Hematology Oncology, Lehigh Valley Hospital Pocono, East Stroudsburg, Pennsylvania, USA; 8 Division of Hematologic Malignancies and Cellular Therapy, Duke University, Durham, North Carolina, USA; 9 Department of Pathology, Duke University, Durham, North Carolina, USA

**Keywords:** cancer, epidemiologic study, race, surveillance, venous thromboembolism

## Abstract

**Background:**

Cancer-associated venous thromboembolism (CA-VTE) represents a major cause of morbidity and mortality in patients with cancer. Despite poor outcomes, there is an ongoing knowledge gap in epidemiologic data related to this association.

**Objectives:**

To compare venous thromboembolism (VTE) characteristics, risk factors, and outcomes between patients with and without active cancer in a racially diverse population.

**Methods:**

Our surveillance project occurred at the 3 hospitals in Durham County, North Carolina, from April 2012 through March 2014. Electronic and manual methods were used to identify unique Durham County residents with VTE.

**Results:**

We identified 987 patients with VTE during the surveillance period. Of these, 189 patients had active cancer at the time of their VTE event. Patients with CA-VTE were older (median age: 69 years vs 60 years, *P* < .0001) and had a lower body mass index (median body mass index: 26.0 kg/m^2^ vs 28.4 kg/m^2^, *P* = .0001) than noncancer patients. The most common cancers in our cohort were gastrointestinal, breast, genitourinary, and lung. The proportion of VTE cases with pulmonary embolism (PE) was greater in the cancer cohort compared with that in the noncancer cohort (58.2% vs 44.0%, *P* = .0004). Overall survival was lower in the CA-VTE group than in patients without cancer (*P* < .0001). Black patients with CA-VTE had lower proportion of PE (52.3% vs 67.1%, *P* = .05) but had decreased survival (*P* < .0003) in comparison with White patients.

**Conclusion:**

Future studies may be needed to continue to evaluate local and national VTE data to improve VTE prevention strategies and CA-VTE outcomes.

## Essentials

• Cancer-associated venous thromboembolism (CA-VTE) in multiracial populations is less understood.
• We studied a racially diverse population with thrombosis with and without active cancer.
• Patients with CA-VTE were older and had a lower body mass index and reduced survival than those without cancer.
• Black individuals with CA-VTE had decreased survival compared with White individuals with CA-VTE.

## 1 Introduction

Cancer-associated venous thromboembolism (CA-VTE) is a common complication and an important cause of morbidity and mortality in patients with cancer. The annual incidence of venous thromboembolism (VTE) overall ranges between 1.37 and 2.47 individuals per 1000 each year [[1], [2], [3], [4], [5], [6]]. When specifically looking at VTE in cancer cohorts, the annual incidence of VTE depends on the primary cancer type. For example, annual incidence rates can range from 1% to 2% for prostate and breast cancers, while it can be as high as 10% for pancreatic cancer [7,8]. Most importantly, CA-VTE incidence rates have continued to rise over the last 2 decades [9], while overall incidence rates of cancer have slowly decreased over this same timeframe [10]. This finding can be related to improving overall survival benefits seen with novel cancer-directed therapies and advances with high-resolution imaging modalities leading to additional unexpected VTE diagnosis [11].

The incidence of CA-VTE is additionally influenced by an interplay of patient-related risk factors [12]. For example, when taking account of the race and ethnicity of the patient population, CA-VTE rates widely vary. Previous literature by Chew et al. [13] noted that Asian/Pacific Island patients have lower risk of development of CA-VTE than White patients in prostate, breast, lung, colorectal, pancreas, and gastric cancers and non-Hodgkin lymphomas. Raskob et al. [14] studied a racially diverse population in Oklahoma County, Oklahoma, and noted highest rates of CA-VTE in non-Hispanic Black patients. Similar to incidence rates, mortality rates for CA-VTE depend upon both patient- and cancer-related risk factors [8,[15], [16], [17], [18]]. Previous literature has documented that the risk of CA-VTE varies based on primary cancer site, stage, duration of time since cancer diagnosis, cancer-directed therapies, and patient-related risk factors [[19], [20], [21], [22]]. Studies have illustrated that the most prothrombotic primary cancer sites are pancreatic, gastric, ovarian, and primary brain tumors [7,8,23]. Various pathways have been proposed to explore the mechanisms between the primary site of cancer and VTE [22,24]. The association is a result of certain tumors expressing procoagulant proteins such as tissue factor, cancer procoagulant, and plasminogen activator inhibitor-1 [9,22,24]. Other tumors may alter host inflammatory response systems through inflammatory cytokines, proangiogenic growth factors, and platelet aggregation and activation agonists leading to a prothrombotic state [9,22,24]. Additional studies have shown that the highest risk of VTE is at the time of cancer diagnosis and in patients with more advanced stage of cancer [[25], [26], [27]]. Finally, certain cancer-directed therapies themselves, as well as the means to deliver treatment, such as central venous catheters, are known to be prothrombotic [[28], [29], [30]]. Ultimately, CA-VTE is associated with high mortality, up to 3-fold greater than noncancer VTE counterparts [8,[15], [16], [17], [18]].

We investigated VTE rates, risk factors, treatment patterns, and outcomes in Durham County, North Carolina, between the years 2012 and 2014 [6]. Within this racially diverse cohort, we have expanded this analysis by evaluating clinical differences among patients with a history of VTE with and without active cancer. Through a meticulous surveillance program, we are able to contribute to the limited epidemiologic data outlining the association of cancer and VTE in populations with racial heterogeneity.

## 2 Methods

We identified patients with a history of VTE at Duke University Hospital, Duke Regional Hospital, and the Durham Veterans’ Administration Medical Center within Durham County, North Carolina. Patients were included if they resided within Durham County at the time of diagnosis of VTE between April 1, 2012, and March 31, 2014 [6]. Deep venous thrombosis (DVT) and pulmonary embolism (PE) were searched by International Classification of Diseases, Ninth Revision (ICD-9) and Current Procedural Terminology (CPT) codes during the surveillance period. A detailed description of the surveillance strategy, including a detailed report on the relevant ICD-9 and CPT codes, has been previously published [31]. All cases identified by electronic medical record screening were confirmed by review of imaging studies, autopsy reports, or clinical records by trained research staff and clinicians. Consistent with our previous paper, patients living outside Durham County at time of diagnosis, or at time of death for autopsy cases, were not included [6].

Data investigated included demographic information such as patient age, sex, self-reported race and ethnicity, and body mass index (BMI), with obesity classified as a BMI of greater than 30 kg/m^2^. Past medical history of comorbidities was additionally obtained including thrombosis history. Data on provoking risk factors, thromboprophylaxis at time of event, and type of anticoagulation prescribed for treatment were also collected. Additional data abstracted included cancer type, stage, certain cancer-directed therapies, and patient outcomes including recurrent thrombosis, major hemorrhage, and death (all-cause mortality). We also searched public records from the Durham County Register of Deeds/Vital Records Office on all patients without follow-up documented in the electronic medical records to identify individuals who had died.

The definition of DVT included all thrombotic events involving the inferior vena cava; the deep veins of the pelvis and proximal and distal lower extremities; the superior vena cava; and the deep veins of the upper thorax, neck, and upper extremities. PE included all thromboembolic events involving the pulmonary artery and its branches. Prior DVT and/or PE were defined as occurring before the surveillance period. Inpatient VTE was defined as VTE events that occurred after 2 days of hospital admission. Recurrent VTE was defined as a new DVT and/or PE or clear demonstration of thrombosis extension on a subsequent imaging study. Recurrent VTE included both during the same hospitalization as the index event or at a completely different timepoint. Recent hospitalization was defined as occurring within 90 days of the VTE event. Similarly, surgery was considered a provoking risk factor if within 90 days of the VTE event. Major surgery was defined as a surgery or procedure that lasted 60 minutes or more, and minor surgery lasted less than 60 minutes. VTE that occurred in the setting of active cancer was classified as CA-VTE. Active cancer was defined as having a positive pathology report for cancer and/or current evidence of disease within the last 6 months (based on imaging, biopsy, etc.) regardless of whether they were on cancer-directed therapies. Both solid tumors and hematologic cancers were included and grouped based upon the site of primary disease. Information regarding prior history of cancer that was not currently active was not collected. A patient was considered a smoker if they admitted to current smoking at time of VTE diagnosis. All data were reviewed by research staff for accuracy, duplicate entries were removed, and final data were entered into a REDCap database before analysis [6]. This population-based surveillance study, a public health activity performed according to guidance from the CDC and the US Department of Health and Human Services, was deemed exempt from review by the Duke Institutional Review Board, and patient informed consent was waived.

Summary statistics, medians/ranges for continuous variables, and counts/percentages for categorical variables were used to summarize data. Comparison between groups and continuous variables was conducted using Wilcoxon’s test, and associations between any categorical variables were examined using chi-squared tests or Fisher’s exact test. Statistical comparisons between cohorts required at least 5 values within each cohort to calculate a *P* value. No adjustment was made for multiple testing. Statistical significance was examined at alpha = 0.05. In addition, Kaplan–Meier curves were used to examine the difference in survival between the cancer and noncancer groups separately within Black and White race and survival by race (Black cohort and White cohort) within cancer and noncancer groups. Statistical analyses were performed using SAS 9.4 statistical software (SAS Institute Inc).

## 3 Results

### 3.1 Demographics

We identified a total of 987 patients with VTE during the surveillance period. Of these, 189 patients had active cancer at the time of diagnosis of their VTE event. Demographic characteristics of our cohort are described (Table 1). Distribution based on sex was similar between the groups with and without cancer. Patients with CA-VTE were older than the noncancer group (median age: 69 years vs 60 years, *P* < .0001). While overall there was no difference in race for the cancer groups (*P* = .30), in a subgroup analyses, there was a slight difference in the proportion of White patients in the noncancer and CA-VTE groups (47.5% vs 38.5%, *P* = .03) as well as of the Black patients (47.7% vs 58.7%, *P* = .03) in the cancer groups. In addition, the proportion of Black patients with cancer (21.9%; 111/508) was higher than that of White patients (16.2%; 72/452), which was statistically significant (*P* = .03). Data on BMI were available for 857 patients. Patients with CA-VTE had a lower BMI when compared with their noncancer counterparts (median BMI: 26.0 kg/m^2^ vs 28.4 kg/m^2^, *P* = .0001). The prevalence of other medical comorbidities including diabetes mellitus, heart disease, congestive heart failure, hypertension, peripheral arterial disease, and renal disease was comparable between the 2 groups (Table 1).

**Table 1:** Baseline characteristics of patients without and with active cancer diagnosed with deep venous thrombosis or pulmonary embolism.

Characteristic	No cancer (*n* = 798)	Cancer (*n* = 189)	*P* value
Sex, female	409 (51.3%)	103 (54.5%)	.42
Age, y (Q1, Q3)	60 (45, 74)	69 (58, 77)	<.0001
Race			.30
White	379 (47.5%)	73 (38.6%)	.03
Black	397 (49.7%)	111 (58.7%)	.03
Asian	3 (0.4%)	1 (0.5%)	
Native Hawaiian/Pacific Islander	3 (0.4%)	0 (0.0%)	
American Indian/Alaskan Native	1 (0.1%)	0 (0.0%)	
Unknown	15 (1.9%)	4 (2.1%)	
BMI, kg/m^2^ (*N* = 857), median (Q1, Q3)	28.4 (23.6, 35)	26 (22.5, 30.1)	.0001
Medical comorbidities			
Diabetes mellitus	211 (26.4%)	38 (20.1%)	.07
Heart disease	147 (18.4%)	28 (14.8%)	.24
Congestive heart failure	88 (11.0%)	14 (7.4%)	.14
Hypertension	462 (57.9%)	120 (63.5%)	.16
Peripheral arterial disease	24 (3.0%)	8 (4.2%)	.39
Pulmonary disease	190 (23.8%)	50 (26.5%)	.45
Kidney disease	95 (11.9%)	21 (11.1%)	.76

BMI, body mass index; DVT, deep venous thrombosis; PE, pulmonary embolism.

### 3.2 Cancer characteristics by race

The most common cancers in our cohort with CA-VTE were gastrointestinal, breast, genitourinary, and lung (Table 2 and Supplementary Table S1). Prevalence of genitourinary cancers was higher in Black patients in comparison with White patients (25.2% vs 9.6%, *P* = .009; Table 2). There was no statistically significant difference in the prevalence of other cancers between the Black and White patient cohorts. Metastatic disease was identified in 56 of 77 Black patients for whom data are available (72.7%), in comparison with 37 of 57 White patients (64.9%, *P* = .33). Use of cancer-directed therapy at the time of diagnosis of VTE was greater in the White patient cohort in comparison with the Black patient cohort (78.3% vs 60.2%, *P* = .02).

**Table 2:** Cancer characteristics in Black and White individuals with cancer-associated venous thromboembolism.

Cancer characteristic	Black (*n* = 111)	White (*n* = 73)	Total (*n* = 189)	*P* value
Primary cancer site				
Gastrointestinal	28 (25.2%)	11 (15.0%)	39 (20.6%)	.10
Genitourinary	28 (25.2%)	7 (9.6%)	35 (18.5%)	.009
Lung	21 (18.9%)	20 (27.4%)	42 (22.2%)	.18
Breast	11 (9.9%)	12 (16.4%)	23 (12.5%)	.19
Gynecologic	10 (9.0%)	6 (8.2%)	18 (9.5%)	.81
Hematologic	9 (8.1%)	5 (6.8%)	15 (7.9%)	.74
Sarcoma	3 (2.7%)	2 (2.7%)	5 (2.7%)	
Brain	0 (0.0%)	2 (2.7%)	3 (1.5%)	
ENT	2 (1.8%)	5 (6.8%)	7 (3.8%)	
Other	2 (1.8%)	3 (4.1%)	5 (2.6%)	
Metastatic cancer	56/77 (72.7%)	37/57 (64.9%)	95/138 (68.8%)	.33
Cancer therapy at the time of VTE diagnosis	56/93 (60.2%)	47/60 (78.3%)	105/156 (67.3%)	.02

Distribution of specific subtypes of gastrointestinal and genitourinary cancers is described in Supplementary Table S1. The other category includes carcinoid, neuroendocrine tumors, small cell cancer, and squamous cell cancer of unknown primary, paraganglioma, and thyroid cancer. Three individuals had more than 1 primary site of cancer. Cancer diagnoses were reviewed by the study investigators for accuracy and consistency. Prevalence of metastatic cancer and use of cancer therapy is described for individuals for whom these data were collected.

ENT, ear, nose, throat; VTE, venous thromboembolism.

### 3.3 VTE characteristics

The proportion of VTE cases with PE was greater in the cancer cohort compared with the proportion of patients with PE without cancer (58.2% vs 44.0%, *P* = .0004; Table 3), but clinical symptoms of PE were more common among those with PE in the noncancer group (94.3% vs 87.3%, *P* = .01). Within the cancer group, proportion of PE was greater in the White patient cohort in comparison with the Black patient cohort (67.1% vs 52.3%, *P* = .05; Supplementary Table S2). The distribution for location of DVT was comparable between the cancer and noncancer cohorts with the exception of a greater proportion of left leg DVT in the noncancer patients (28.6% vs 16.9%, *P* = .001; Table 3).

**Table 3:** Characteristics of the venous thromboembolism in patients without and with active cancer.

Characteristic	No cancer (*n* = 798)	Cancer (*n* = 189)	*P* value
Pulmonary embolism	351 (44%)	110 (58.2%)	.0004
PE only diagnosis	264 (33.1%)	85 (45%)	.002
Symptomatic PE	331 (94.3%)	96 (87.3%)	.01
Segmental PE	287 (81.8%)	93 (84.5%)	.50
DVT	534 (66.9%)	104 (55%)	.002
DVT only diagnosis	447 (56%)	79 (41.8%)	.0004
Symptomatic DVT	496 (92.9%)	89 (85.6%)	.01
DVT location^a^			
Left leg	228 (28.6%)	32 (16.9%)	.001
Right leg	177 (22.2%)	43 (22.8%)	.87
Left arm	50 (6.3%)	12 (6.3%)	.97
Right arm	82 (10.3%)	18 (9.5%)	.76

DVT, deep vein thrombosis; PE, pulmonary embolism.

a Patients are included if they have a DVT in the noted location. Individual patients may have had more than one location of DVT on presentation and are included in both cohorts. Due to this, total percentages do not equal 100.

Of the total 987 patients, 167 were diagnosed with VTE as an inpatient. There was no statistically significant difference in proportion of DVT vs PE while inpatient in the 2 groups (Supplementary Table S3). Less than half (45.7% and 44.7%) of inpatients diagnosed with VTE in both cancer and noncancer cohorts were on thromboprophylaxis at the time of the VTE event (Supplementary Table S3).

### 3.4 History of prior VTE

The occurrence of prior VTE was greater in the noncancer cohort. Prior VTE was present in 10.6% of patients in the cancer group compared with 20.4% patients in the noncancer group (*P* = .002; Table 4). No patients in the cancer cohort had >2 prior VTE events as opposed to 40 patients in the noncancer cohort (0% vs 5.0%). Roughly half the patients with prior VTE remained on an anticoagulant for a prior event (VTE and/ or non-VTE event) in both cancer and noncancer cohorts.

**Table 4:** History of prior venous thromboembolism and anticoagulant use in patients without and with active cancer.

History of VTE	No cancer (*n* = 798)	Cancer (*n* = 189)	*P* value
History of prior VTE	163 (20.4%)	20 (10.6%)	.002
1 prior VTE event	123 (15.4%)	20 (10.6%)	.02
>2 prior VTE events	40 (5.0%)	0 (0.0%)	
Antithrombotic use at time of VTE	260 (32.6%)	51 (27%)	.10
Anticoagulant use for prior event (VTE and non-VTE)	84 (10.5%)	10 (5.3%)	.03
Enoxaparin/heparin/warfarin	82 (10.3%)	7 (3.7%)	.005
Antiplatelet use for prior event (VTE and non-VTE)	196 (24.6%)	43 (22.8%)	.60

VTE, venous thromboembolism.

### 3.5 Risk factors for VTE

There was no difference in the proportion with recent major or minor surgery between the cancer and noncancer cohorts (Table 5). Recent hospitalization was more prevalent in the cancer population (44.0% vs 34.7%, *P* = .03). The percentage of patients with obesity in the cancer cohort was less than that in the noncancer cohort (23.8% vs 51.8%, *P* < .0001). Central venous catheter–associated upper extremity DVT was similar between the groups (4.0% vs 5.7%, *P* = .39). A smaller proportion of patients in the cancer cohort had extended travel of >4 hours compared with the noncancer cohort (2.6% vs 8.5%, *P* = .01). No patients in the cancer group were on hormonal contraception compared with 36 patients in the noncancer group (0% vs 6.1%). There were no statistically significant differences in hormone replacement, pregnancy, or postpartum period between the groups. Smoking was less common in those with cancer than in those in the noncancer group (13.2% vs 20.6%, *P* = .04).

**Table 5:** Risk factors for outpatient venous thromboembolism in patients without and with active cancer.

Risk factor	No cancer (*n* = 669)	Cancer (*n* = 151)	*P* value
Transient risk factors			
Recent surgery	134 (22.6%)	33 (21.9%)	.84
Major surgery	106 (79.1%)	24 (72.7%)	.49
Minor surgery	26 (19.4%)	9 (27.3%)	.25
Recent hospitalization	205 (34.7%)	66 (44%)	.03
Central venous catheter in affected arm with DVT	34 (5.7%)	6 (4.0%)	.39
Fracture	30 (5.1%)	8 (5.3%)	.90
Extended travel (>4 h)	50 (8.5%)	4 (2.6%)	
Hormone use/pregnancy/medications			
Hormonal contraceptive	36 (6.1%)	0 (0.0%)	
Hormone replacement	3 (0.5%)	0 (0.0%)	
Pregnancy	9 (1.5%)	0 (0.0%)	
Postpartum period	3 (0.5%)	0 (0.0%)	
SERM use (eg, tamoxifen)	1 (0.2%)	1 (0.7%)	
Corticosteroids	47 (7.9%)	12 (7.9%)	.99
Persistent nonsurgical risk factors			
Prolonged/permanent immobility	151 (25.5%)	28 (18.5%)	.07
Active autoimmune disease	59 (9.9%)	15 (9.9%)	.99
Transvenous pacemaker	14 (2.4%)	1 (0.7%)	
BMI > 30 kg/m^2^	307 (51.8%)	36 (23.8%)	<.0001
Smoking	122 (20.6%)	20 (13.2%)	.04

BMI, body mass index; DVT, deep vein thrombosis; SERM, selective estrogen receptor modulator.

### 3.6 Patterns of anticoagulant use for treatment

Enoxaparin use as the sole anticoagulant was significantly higher in the cancer cohort compared with the noncancer group (61.4% vs 18.3%, *P* < .0001; Supplementary Table S4), and warfarin use was significantly higher in the group without cancer (22.8% vs 60.4%, *P* < .0001). Inferior vena cava filter placement was performed in 6.3% of cancer VTE patients and 7.1% of noncancer VTE patients (*P* = .78). No significant differences were noted in the use of other interventions (Supplementary Table S4).

### 3.7 Outcomes

**Figure 1:**
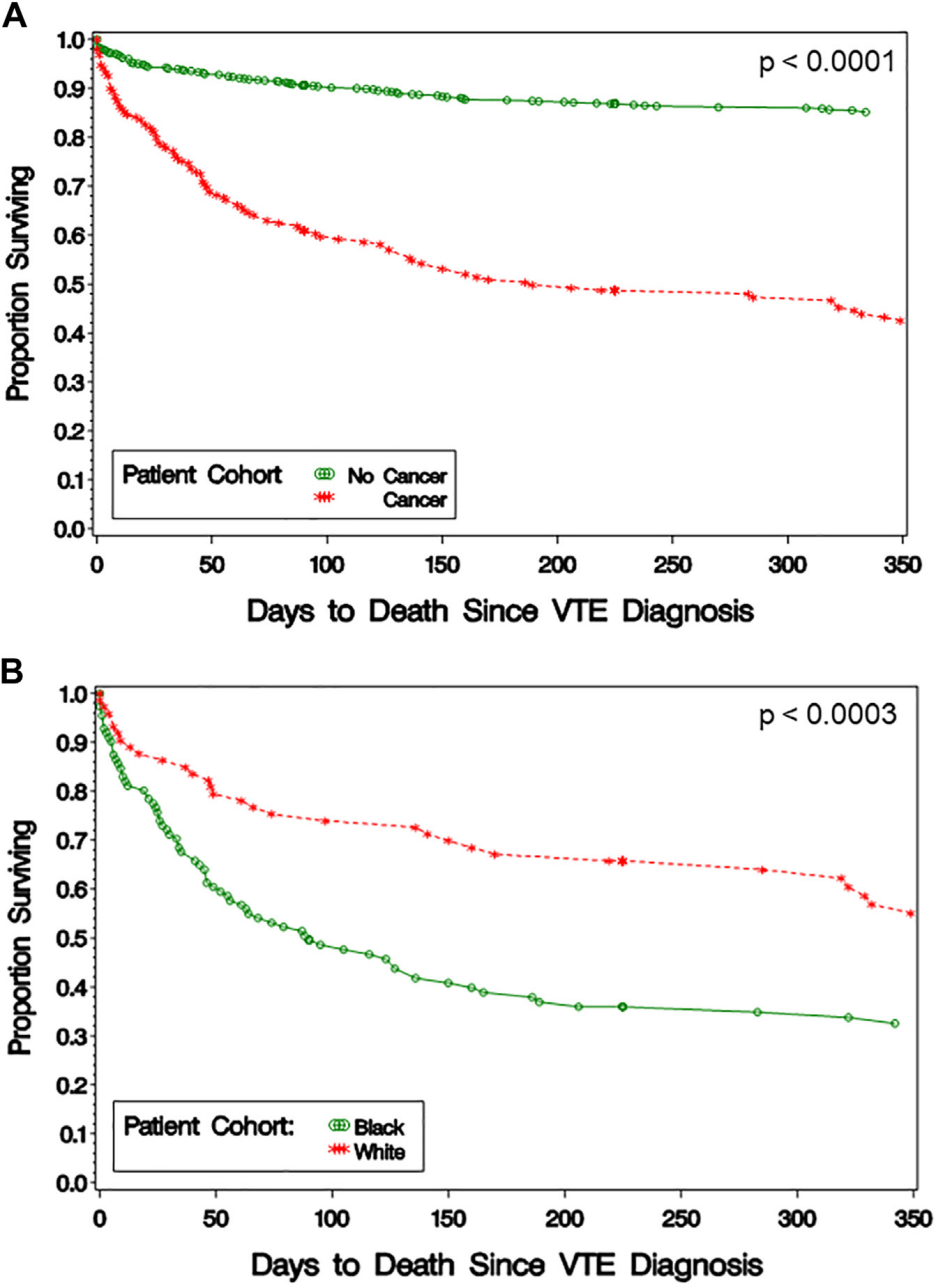
Kaplan–Meier plot depicting (A) proportion of patients with VTE without and with cancer who survived, by day from VTE diagnosis, and (B) proportion of patients with CA-VTE who survived, by race, in respective Black and White cohorts. CA-VTE, cancer-associated venous thromboembolism; VTE, venous thromboembolism.

The most frequent adverse clinical outcome following VTE was death, which occurred in 107 patients with VTE and cancer (56.6% of 189 patients) and 117 patients with VTE but without cancer (14.7% of 798 patients; Table 6). The proportion of patients surviving was higher for patients without cancer within the first week of VTE diagnosis and the differences continued to increase over the next 12 months (*P* < .0001; Figure 1A). Survival in the CA-VTE cohort was lower in Black individuals in comparison with White individuals (*P* < .0003; Figure 1B). There was no significant difference in survival between Black and White patients with noncancer-associated VTE (*P* = .06; Supplementary Figure S1). Mortality in patients with DVT only in the CA-VTE cohort was higher in Black individuals than in White individuals (9.4% vs 0.0%, *P* = .32). Recurrent VTE appeared to occur more frequently in the noncancer group, but the number of events was small, particularly in the CA-VTE group (Table 6). Major bleeding events occurred in similar proportions of patients with or without cancer (Table 6).

**Table 6:** Recurrent venous thromboembolism, major bleeding, and deaths in patients with and without cancer.

Time interval since VTE diagnosis				
Outcome	0-7 d	8 d-6 mo	6-12 mo	Total
Cancer patients (189 at baseline)				
Recurrent VTE, *n* (events)	3 (3)	7 (8)	1 (1)	11 (12)
Major bleeding, *n* (events)	5 (5)	9 (10)	1 (1)	15 (16)
Deaths	19	73	15	107
Lost to follow-up	-	5	17	22
Noncancer patients (798 at baseline)				
Recurrent VTE, *n* (events)	22 (22)	40 (46)	18 (23)	80 (91)
Major bleeding, *n* (events)	17 (17)	22 (24)	10 (11)	49 (52)
Deaths	23	75	19	117
Lost to follow-up		52	120	172

VTE, venous thromboembolism.

## 4 Discussion

The relationship between cancer and VTE has been well documented [9,22]. CA-VTE takes a significant toll given further decline in quality of life, increased healthcare costs, interruption of cancer-directed therapy given bleeding risk, increased risk of recurrent VTE, and ultimately mortality [9]. However, there is an ongoing knowledge gap in epidemiologic data outlining this association in populations with racial heterogeneity [9,14]. In recent years, there have been increasing data from population-based studies on incidence of CA-VTE from California, Oklahoma, and a nationwide VA study to address these concerns [13,14,32,33]. Previous literature denotes that non-Hispanic Black patients have a higher incidence of CA-VTE whereas Asian/Pacific Islander people have a lower incidence of CA-VTE [13,14,32,33]. Genomic factors as well as socioeconomic determinants are plausible explanations for this discrepancy [32]. These previous studies have enhanced our understanding of incidence of CA-VTE in racially diverse populations and reinforced the decreased survival seen in patients with CA-VTE as opposed to cancer without VTE and noncancer VTE [13,14]. However, a potential pitfall of some of the previous literature is the sole utilization of ICD-9 or ICD-10 codes for screening for VTE allowing for inaccuracy [13,32]. Our study used a meticulous surveillance program to explore VTE characteristics, anticoagulation practices including prophylaxis use, risk factors, and outcomes of CA-VTE as compared with noncancer VTE in the general population in Durham County, North Carolina.

Our total cohort consisted of 987 patients of which 19% had active cancer at the time of diagnosis of VTE. Durham County, North Carolina, is a racially diverse county with a population estimate of 267,587 people in the census of 2010 [34]. This population consisted of 43.6% non-Hispanic White people, 35.3% non-Hispanic Black people, 6.0% Asian/Pacific Island people, and a smaller percentage of multiracial, unknown race/ethnicity, or other racial/ethnic populations [34]. Therefore, Durham County is home to a more racially diverse population in comparison with the overall US national population based on 2010 and 2020 Census data [35]. Of note, of all the epidemiologic studies on CA-VTE in racially heterogenous populations, Durham County has the greatest proportion of Black people [13,14,[32], [33], [34]]. Our total cohort with VTE had a higher number of Black patients (51.5%) with smaller percentages of White patients (45.8%) and Asian/Pacific Island patients (0.4%) compared with those in the county population data [6,34]. Of the 189 patients with active cancer at time of VTE, 58.7% were Black patients compared with 38.6% White patients, and a smaller percentage (2.7%) were either Asian/Pacific Island patients or multiracial patients or their race/ethnicity was unknown. In our study, patients with active cancer when diagnosed with VTE were more likely to be older and have a lower BMI (Table 1). Advancing age is an important risk factor for cancer and patients with cancer can develop a syndrome of cachexia resulting in lower BMI [36,37].

The most common cancers in our CA-VTE cohort were gastrointestinal, genitourinary, lung, and breast, which is similar to what has been reported previously [14]. This was also consistent with documented cancer incidence rates in Durham County from 2010 to 2014, when the highest rates of cancer were breast and prostate, followed by lung/bronchus and finally colorectal [38]. When categorized by race, our study demonstrated that Black patients with CA-VTE had a statistically significant higher percentage of genitourinary cancers compared with White patients. This was again consistent with county-wide data from 2014 to 2018, with Black patients noted to have the higher incidence of prostate cancer compared with White patients (144.4 per 100,000 people vs 97.4 per 100,000 people) in Durham County [39]. The majority of patients with CA-VTE for whom we had data had documented metastatic disease when diagnosed with VTE (Table 2). This finding is consistent with previous literature demonstrating higher incidence of VTE with metastatic disease compared with localized disease [27,[40], [41], [42]]. This association may be due to extrinsic compression of vascular structures from the cancer itself, or potentially due to CA-VTE indicating a more aggressive disease seen in patients with metastatic disease [27,43]. For patients with available data, we noted a statistically significant difference with White patients more often on cancer-directed therapy at time of VTE diagnosis compared with Black patients (78.3% vs 60.2%, *P* = .02). Plausible explanations for this could include a more palliative approach in Black patients given more advanced disease, timing between the diagnosis with cancer and the identification of a VTE, or possibly increased side effects from cancer-directed therapy. Lastly, it is imperative to note that it is certainly possible that Black patients were less often offered systemic treatment compared with White patients. Previous literature has illuminated racial disparities in cancer outcomes, which may have played a role in our findings [[44], [45], [46], [47]]. Ultimately, additional research focused on this topic needs to continue to improve outcomes for all Durham County residents.

Patients with active cancer had more asymptomatic PE compared with patients without active cancer. This likely reflects the staging and surveillance imaging studies performed in the active cancer cohort revealing small or silent PE [48,49]. An increase in the incidence of incidental cancer-associated PE has been documented by others likely due to use of high-resolution imaging for staging and follow-up on disease burden [11]. In contrast to the findings by Datta et al. [32] noting that the racial disparities in VTE incidence were greatest for PE, with the highest incidence being noted in Black patients, our study population had a greater proportion of PE in White individuals with CA-VTE in comparison with Black individuals. This finding may be due to greater use of imaging for staging and/or greater use of imaging for investigation of VTE symptoms resulting in detection of incidental PE in White individuals in our cancer cohort. When comparing patients with cancer with those without cancer, patients without cancer were more likely to have DVT and those with PE were more likely to be symptomatic. This association was likely due to the imaging studies obtained having a high pretest probability and concern for symptomatic thrombosis. Interestingly, we noted a statistically significant increase in left leg DVT in our noncancer cohort compared with other locations, which has been documented by others [50]. Lastly, our data demonstrated that greater than 50% of both cancer and noncancer patients with VTE were not on thromboprophylaxis while inpatient at the time of thrombosis. In the cancer cohort, this may be due to clinical concern of higher bleeding risk in these patients, postoperative time frame when the thrombosis occurred, or potentially due to ongoing cytopenias. Since the time frame of our study, much effort has been put toward increasing hospitalized patient’s VTE prophylaxis and we would anticipate higher prophylaxis currently [51].

Patients with cancer were more often treated with enoxaparin as the only anticoagulant administered, while patients without cancer were more likely to be treated with warfarin or rivaroxaban. These findings can largely be attributed to the time frame in which the surveillance was conducted. In 2012 to 2014, the standard-of-care treatment for CA-VTE was enoxaparin after the results from the Randomized Comparison of Low-Molecular-Weight Heparin versus Oral Anticoagulant Therapy for the Prevention of Recurrent Venous Thromboembolism in Patients with Cancer (CLOT) trial. This landmark study showed superior efficacy in reducing recurrent VTE with low-molecular-weight heparin compared with warfarin [52]. For patients without cancer, we can attribute the higher use of warfarin to the ease of administration of the medication and reduced cost compared with enoxaparin. The low number of patients prescribed either apixaban or rivaroxaban can again be attributed to the time frame of our study. The landmark trials demonstrating DOAC efficacy in this cancer VTE patient population were not published until 2018 and subsequent years [[53], [54], [55], [56]]. The cancer VTE treatment guidelines have since been updated, accounting for increased apixaban and rivaroxaban use by clinicians for CA-VTE [57].

Recurrent VTE, hemorrhage, and death are common complications of CA-VTE. Our outcome data reinforce decreased survival in patients with CA-VTE than those with VTE but no cancer and highlights the remarkably lower survival in Black patients with CA-VTE in comparison with White individuals (Figure 1). Raskob et al. [14] had previously shown that mortality varied with race/ethnicity with a trend toward higher mortality in non-Hispanic Black patients when compared with non-Hispanic White patients. Similar results were recently published by Giorgio et al. [58] investigating a large Medicare cohort of over 125,000 individuals from 2011 to 2019. Their study demonstrated an increased 30-day (7.0 % vs 5.8%) and 1-year (40.8% vs 33.8%) mortality in Black individuals compared with White individuals with CA-VTE [58]. Interestingly, in a multivariable regression analysis done by Martens et al. [33], non-Hispanic Black race/ethnicity was not found to be an independent adverse risk factor for CA-VTE mortality. It is important to note that this study by Martens et al. [33] was based out of the Veterans Affairs health care system of which the authors acknowledge the robust access to healthcare for their cohort. When comparing our study to similar studies with disparities in access to health care, our results were consistent with the literature demonstrating worse mortality outcomes for non-White individuals [14,58]. Our study consisted of the highest proportion of Black individuals, helping to contribute primary literature to an underrepresented patient population. Similar to the papers by both Raskob et al. [14] and Giorgio et al. [58], our decreased mortality outcomes in non-Hispanic White individuals with CA-VTE are likely a result of complex and multifactorial etiologies, of which socioeconomic status and access to health care play a large role. In addition, Black individuals in our study had more extensive stage cancer and less often received cancer-directed therapies, all of which likely increased the risk of worse outcomes for this cohort of patients. Furthermore, White patients with CA-VTE likely had more incidentally found PE and therefore the thrombosis was less likely to contribute toward mortality. When further stratifying by thrombosis type, the trend of increased mortality in Black individuals with CA-VTE was noted regardless of whether they had a PE or only DVT. Lastly, our rate of recurrent VTE appeared comparable between the cancer and noncancer VTE groups, but the rates may have been lower given the numbers of patients who did not return to one of the hospitals in Durham County during the follow-up period and were therefore considered lost to follow-up. Moreover, the VTE recurrence rate in the CA-VTE cohort may be lower due to higher mortality in this group. Rates of recurrent VTE and major bleeding were comparable to rates reported in other studies [59,60].

There are several limitations related to this study. As this is a retrospective study, there are likely confounding variables contributing to the survival, hemorrhagic, and recurrent thrombosis outcomes. We collected data from 2012 to 2014 in Durham County, North Carolina. Since this timeframe, Durham County population has grown to an estimated 324,833 from 267,587 [34]. Most recent census data demonstrate similar percentages of non-Hispanic White persons (43.4% vs 47.0%) and non-Hispanic Black persons (35.9% vs 37.2%). It should be noted, however, that there has been an increase in Asian/Pacific Island persons (5.6% vs 0.5%) when compared with that in 2010 [34]. When comparing cancer incidence rates in Durham County, the most recent data are the previously described 2014-2018 statistics [39]. It should be noted, we did not report data on ethnicity as our electronic medical record has limited self-identification options for ethnicity, which has potential for misrepresentation and underrepresentation. Additionally, since our timeframe, newer cancer-directed therapies and cancer-associated anticoagulants have been approved, which may alter current complication and outcome rates. Although we collected limited data on cancer-directed therapies, there are additional agents that are known to be prothrombotic for which we do not have data.

## 5 Conclusions

Our study utilized a meticulously designed VTE surveillance program in a geographic region with great racial diversity to contribute to the limited literature outlining the association of cancer and VTE while illuminating differences in clinical outcomes when stratified by race. Our study illustrated patient-, tumor-, and cancer-directed therapy risk factors associated with VTE in our county. We were additionally able to outline our county-wide anticoagulation practices for both cancer- and non–CA-VTE and document patient-related outcomes. Continued surveillance efforts will be instrumental in future VTE prevention strategies, including prophylaxis practices, for improvement of future CA-VTE outcomes. Further studies and additional investigations may be needed to continue to evaluate county and national VTE data to better understand more recent cancer VTE rates and outcomes.
