# Supplementary material for: An epidemiologic study comparing cancer- and noncancer-associated venous thromboembolism in a racially diverse Southeastern United States county
Source: Res Pract Thromb Haemost. 2024 Apr 26;8(4):102420. doi: 10.1016/j.rpth.2024.102420 (PMC11137544; doi:10.1016/j.rpth.2024.102420)
Supplement: Supplementary material [file mmc1.docx]

An Epidemiological Study Comparing Cancer- and Non-Cancer-Associated Venous Thromboembolism in A Racially Diverse Southeastern United States County

**SUPPLEMENTARY MATERIAL**

**Authors**: Andrew M. Peseski*^1^, Sargam Kapoor*^1^, Maragatha Kuchibhatla^2^, Alys Adamski^3^, Karon Abe^3^, Michele G. Beckman^3^, Nimia L. Reyes^3^, Lisa C. Richardson^3^, Ibrahim Saber^4^, Ryan Schulteis^5^, Bhavana Pendurthi Singh^6^, Andrea Sitlinger^7^, Elizabeth H. Thames^1^, and Thomas L. Ortel^1,8^

*co-first authors

**Affiliations**: ^1^Division of Hematology, Department of Medicine, Duke University, Durham, NC, USA; ^2^Department of Biostatistics and Bioinformatics, Duke University, Durham, NC, USA; ^3^Centers for Disease Control and Prevention, Atlanta, GA, USA; ^4^AbbVie, Inc, North Chicago, IL, USA; ^5^Department of Medicine, Duke University, Durham, NC, USA; ^6^Lehigh Valley Hospital Pocono, East Stroudsburg, PA, USA; ^7^Division of Hematologic Malignancies and Cellular Therapy, Duke University, Durham, NC, USA; ^8^Department of Pathology, Duke University, Durham, NC, USA

**Supplementary Table Legends**

**Supplementary Table S1.** Cancer subtype characteristics in the group of gastrointestinal and genitourinary type of cancers in the Black and White populations.

**Supplementary Table S2**. Characteristics of VTE in patients without and with active cancer by race. Abbreviations used: PE- pulmonary embolism, DVT- deep vein thrombosis

**Supplementary Table S3.** Characteristics of hospitalized patients with VTE without and with cancer

**Supplementary Table S4.** Description of VTE treatments, specifically, anticoagulation, in patients without and with active cancer. Abbreviations used: VTE- venous thromboembolism, IVC- inferior vena cava

**Supplementary Figure Legend**

**Supplementary Figure S1.** Kaplan-Meier plot depicting proportion of patients with non-cancer associated VTE who survived, by race, in respective Black (green line) and White (red line) cohorts.

| GI and GU cancer subgroup characteristic | Black (n=56) | White (n=18) | Total  (n=74) |
| --- | --- | --- | --- |
| Gastrointestinal | 28 (25.2%) | 11 (15.0%) | 39 (20.6%) |
| Pancreatobiliary | 8 (7.2%) | 4 (5.4%) | 12 (6.5%) |
| Liver | 3 (2.7%) | 3 (4.1%) | 6 (3.2%) |
| Gastroesophageal | 3 (2.7%) | 1 (1.3%) | 4 (2.17%) |
| Colorectal | 12 (10.8%) | 3 (4.1%) | 15 (8.1%) |
| Anal | 2 (1.8%) | 0 (0.0%) | 2 (1.0%) |
| Genitourinary | 28 (25.2%) | 7 (9.6%) | 35 (18.5%) |
| Renal | 6 (5.4%) | 1 (1.3%) | 7 (3.7%) |
| Bladder | 5 (4.5%) | 0 (0.0%) | 5 (2.7%) |
| Prostate | 17 (15.3%) | 5 (6.8%) | 22 (11.9%) |
| Testicular | 0 (0.0%) | 1 (1.3%) | 1 (0.5%) |

**Supplementary Table S1.** Cancer subtype characteristics in the group of gastrointestinal and genitourinary type of cancers in the Black and White populations.

|  | Active Cancer | | | No Cancer | | |
| --- | --- | --- | --- | --- | --- | --- |
| Characteristic | Black  (n=111) | White  (n=73) | P-value | Black  (n=397) | White  (n=379) | P-value |
| PE | 58 (52.3%) | 49 (67.1%) | 0.0454 | 188 (47.4%) | 154 (40.6%) | 0.0594 |
| PE only diagnosis | 46 (41.4%) | 38 (52.1%) | 0.1573 | 144 (36.3%) | 113 (29.8%) | 0.0561 |
| Symptomatic PE | 53 (91.4%) | 42 (85.7%) | 0.1937 | 176 (93.6%) | 147 (95.5%) | 0.1171 |
| Segmental PE | 50 (86.2%) | 40 (81.6%) | 0.1955 | 160 (85.1%) | 119 (77.3%) | 0.0098 |
| DVT | 65 (58.6%) | 35 (47.9%) | 0.1573 | 253 (63.7%) | 266 (70.2%) | 0.0561 |
| DVT only diagnosis | 53 (47.7%) | 24 (32.9%) | 0.0455 | 209 (52.6%) | 225 (59.4%) | 0.0594 |
| Symptomatic DVT | 56 (86.2%) | 30 (85.7%) | 0.2134 | 233 (92.1%) | 249 (93.6%) | 0.0442 |
| DVT location |  |  |  |  |  |  |
| Left leg | 18 (16.2%) | 13 (17.8%) | 0.7777 | 107 (27.0%) | 119 (31.4%) | 0.1729 |
| Right leg | 31 (27.9%) | 12 (16.4%) | 0.0716 | 79 (19.9%) | 92 (24.3%) | 0.1416 |
| Left arm | 5 (4.5%) | 5 (6.8%) | 0.4924 | 23 (5.8%) | 25 (6.6%) | 0.6429 |
| Right arm | 11 (9.9%) | 5 (6.8%) | 0.4710 | 40 (10.1%) | 40 (10.6%) | 0.8265 |

**Supplementary Table S2**. Characteristics of VTE in patients without and with active cancer by race. Abbreviations used: PE- pulmonary embolism, DVT- deep vein thrombosis.

| Characteristic | No cancer (n=129) | Cancer (n=38) | P-value |
| --- | --- | --- | --- |
| PE | 36 (27.9%) | 14 (36.8%) | 0.2905 |
| DVT | 99 (76.7%) | 26 (68.4%) | 0.2987 |
| DVT only | 93 (72.1%) | 24 (63.2%) | 0.2905 |
| Days from admission until VTE [Mean (SD)] | 11.6 (11.2) | 10.6 (9.9) | 0.7815 |
| Thromboprophylaxis at time of VTE | 59 (45.7%) | 17 (44.7%) | 0.9134 |

**Supplementary Table S3**. Characteristics of hospitalized patients with VTE without and with cancer.

| Treatment | No cancer (n=798) | Cancer (n=189) | P-value |
| --- | --- | --- | --- |
| Any anticoagulant |  |  |  |
| Enoxaparin, with or without other anticoagulants | 491 (61.5%) | 147 (77.8%) | <0.0001 |
| Enoxaparin only | 146 (18.3%) | 116 (61.4%) | <0.0001 |
| Warfarin | 482 (60.4%) | 43 (22.8%) | <0.0001 |
| Fondaparinux | 3 (0.4%) | 3 (1.6%) |  |
| Apixaban | 4 (0.5%) | 0 (0.0%) |  |
| Rivaroxaban | 60 (7.5%) | 3 (1.6%) |  |
| Thrombolytic therapy | 18 (2.3%) | 2 (1.1%) |  |
| IVC Filter | 57 (7.1%) | 12 (6.3%) | 0.7665 |
| Thrombectomy/ embolectomy | 6 (0.8%) | 1 (0.5%) |  |

**Supplementary Table S4.** Description of VTE treatments, specifically, anticoagulation, in patients without and with active cancer. Abbreviations used: VTE- venous thromboembolism, IVC- inferior vena cava
