# Supplementary figures and images for: An epidemiologic study comparing cancer- and noncancer-associated venous thromboembolism in a racially diverse Southeastern United States county
Source: Res Pract Thromb Haemost. 2024 Apr 26;8(4):102420. doi: 10.1016/j.rpth.2024.102420 (PMC11137544; doi:10.1016/j.rpth.2024.102420)

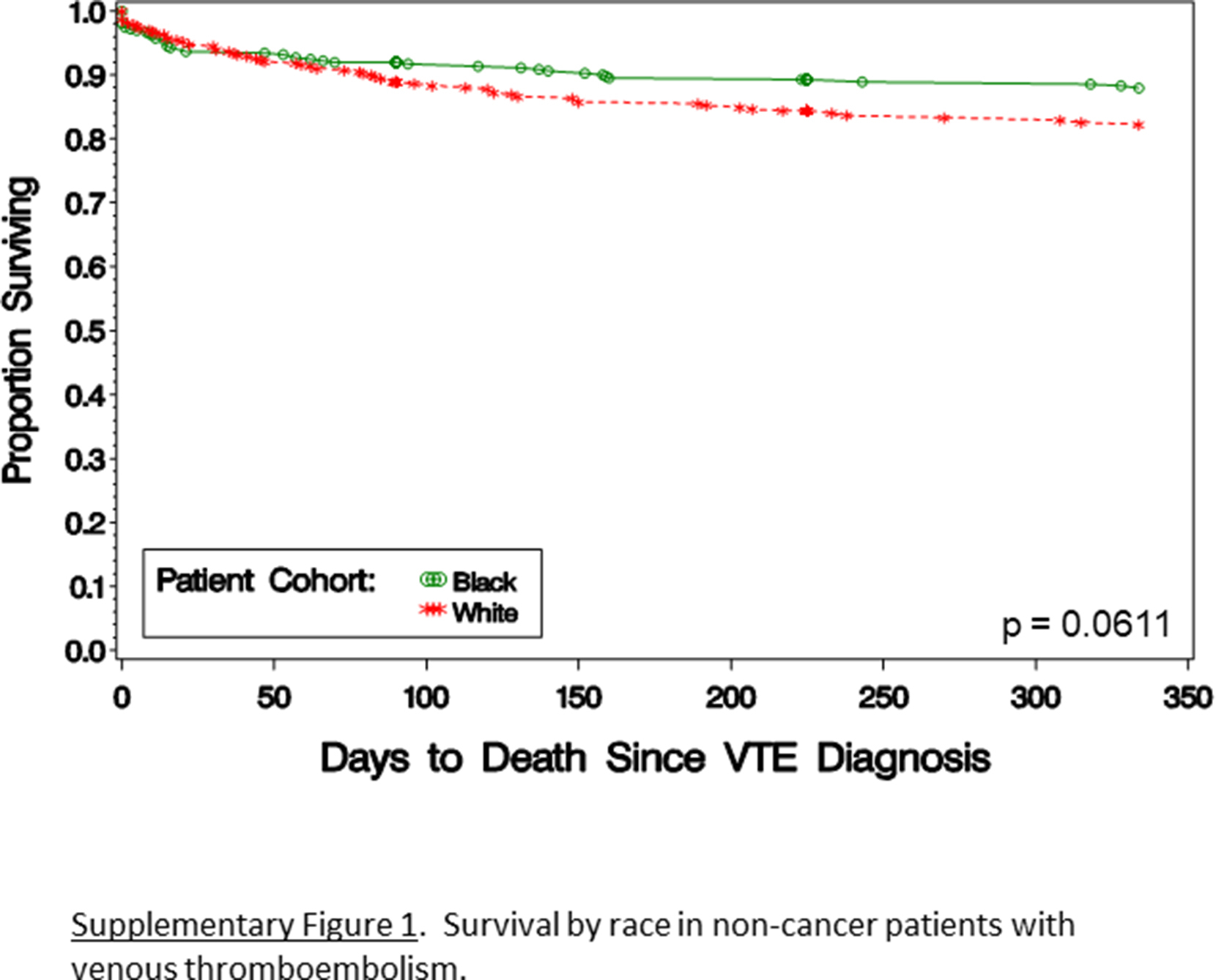

Supplement: Supplemental Figure 1 [file figs1.jpg]
